# Candidiasis as a Potential Marker of Imminent Mortality in Palliative Care: A Retrospective Observational Study

**DOI:** 10.1177/26892820251374977

**Published:** 2026-01-23

**Authors:** Andreia Mandim, Licinia Araújo, Susete Freitas

**Affiliations:** 1 Serviço de Medicina Interna, Unidade Local de Saúde Póvoa de Varzim/Vila do Conde, Vila do Conde, Portugal.; 2 Unidade de Cuidados Paliativos, SESARAM, Madeira, Portugal.

**Keywords:** candidiasis, palliative care, 30-day mortality, corticosteroids, prognostic marker

## Abstract

**Background:**

Predicting short-term mortality in patients receiving palliative care can help tailor interventions, manage expectations, and improve end-of-life planning. This study explores whether candidiasis, particularly in patients on corticosteroids, is associated with increased 30-day mortality.

**Methods:**

We conducted a retrospective study of patients admitted to a specialized palliative care unit in 2022. Data collected included demographics, candidiasis diagnosis (oral/esophageal), corticosteroid use, and mortality. Patients with candidemia were excluded. Of 59 deaths in the unit, 35 had complete records and met inclusion criteria.

**Results:**

Of the 35 patients analyzed, 71.4% were female; the median age was 61. Ten patients developed candidiasis, all with advanced cancer and functional decline. Among candidiasis cases, 30-day mortality was 60% versus 0% in those without. Median time from candidiasis diagnosis to death was 19 days. Among corticosteroid users, candidiasis was associated with significantly higher short-term mortality.

**Conclusions:**

Candidiasis in patients receiving palliative care—particularly those on corticosteroids—may indicate imminent death. These findings suggest its potential as a simple prognostic marker in resource-limited settings, meriting further prospective validation.

## Introduction

Patients receiving palliative care often experience rapid clinical deterioration. Predicting short-term mortality can help tailor interventions, manage expectations, and improve end-of-life planning. Candidiasis, a common opportunistic infection, may reflect immunosuppression or poor functional status. Previous studies suggest that Candida infections correlate with poor prognosis in advanced illness, but data in specialist palliative care settings remain limited. Our unit admits patients with advanced, life-limiting illnesses for symptom control or end-of-life care—two categories that may differ in prognosis and treatment approach. This study investigates whether candidiasis is associated with increased 30-day mortality in such a population.

## Methods

We conducted a retrospective observational study in the 10-bed inpatient palliative care unit at Serviço de Saúde da Região Autónoma da Madeira (SESARAM), Portugal, from January to December 2022. The unit admitted 224 patients in 2022, with an average length of stay of 9 days. Inclusion criteria were adult patients admitted to the unit who died in 2022 with complete electronic records. Of 59 deaths, 35 met these criteria.

Definitions: Candidiasis referred to oral or esophageal mucosal infection diagnosed clinically and, where applicable, confirmed microbiologically. Candidemia, defined as isolation of Candida species from blood cultures, was not observed in this cohort. ‘Systemic fungal infection’ refers to disseminated disease with organ involvement, which was absent in our patients.

Data collected included demographics, underlying diagnosis (oncologic—solid or hematological—vs. nononcologic), corticosteroid use, presence and site of candidiasis, date of diagnosis, and date of death. Corticosteroid use was defined as systemic administration for ≥5 days.

The outcome was 30-day mortality from candidiasis diagnosis. Length of stay from admission to death was calculated for the whole cohort and for candidiasis and noncandidiasis subgroups.

This study was reviewed by the Institutional Review Board of SESARAM, Portugal, which waived the requirement for formal approval because the research was a retrospective review of anonymized records.

## Results

Of 35 patients, 25 (71.4%) were female. The median age was 61 years. All had advanced malignancy, predominantly gastrointestinal and gynecological cancers; none had hematological malignancies. All patients had Eastern Cooperative Oncology Group Scale (ECOG) ≥ 3 and required full assistance. Ten patients (28.6%) developed candidiasis (8 oral, 2 esophageal), mostly while on corticosteroids (*n* = 9). Median length of stay was 9 days overall, 12 days in candidiasis cases, and 7 days in noncandidiasis cases.

Among candidiasis cases, median time from diagnosis to death was 19 days; 30-day mortality was 60% versus 0% in those without candidiasis. In corticosteroid users, candidiasis was associated with significantly higher short-term mortality (60% vs. 0%). No patients had candidemia or received antifungal prophylaxis.

## Discussion

Candidiasis was associated with markedly higher 30-day mortality in this palliative cohort, supporting the hypothesis that mucosal fungal infections may signal terminal decline. The absence of candidemia highlights the prognostic value of localized candidiasis, particularly in resource-limited settings.

Our prevalence of candidiasis (28.6%) is similar to rates reported in other palliative cohorts,^1,2^ though direct comparisons are limited by heterogeneity in populations and definitions. Our results extend prior findings by focusing on a homogenous, high-dependency advanced cancer population, nearly all on corticosteroids.

Limitations include retrospective design, small sample size, and absence of some prognostic variables (e.g., albumin, performance scales). Future studies should stratify by cancer type (solid vs. hematological), treatment intensity, and goals of care and prospectively evaluate candidiasis as a prognostic tool.

## Conclusion

Candidiasis, especially in patients receiving corticosteroids in a palliative care unit, may indicate imminent death. Recognition of this association could support timely care planning and communication with patients and families. Prospective studies are warranted to validate these findings.
